# New Appliances and Things Medical

**Published:** 1897-06-12

**Authors:** 


### NEW APPLIANCES AND THINGS MEDICAL.

**[We shall be glad to receive, at our Office, 28 & 29, Southampton Street, Strand, London, W.C., from the manufacturers, specimens of all new preparations and appliances which may be brought out from time to time.]**

### NEW PALATINOIDS.

(Oppenheimer, Son, and Co., Limited, London.)

Our attention has lately been directed to a new variety of palatinoid prepared by Messrs. Oppenheimer. The palatinoid contains doses of phosphates of iron, quinine, and strychnine, equivalent to half a drachm of Easton's syrup. This popular preparation in the ordinary form of syrup is prone to undergo decomposition if kept in stock for any length of time; whereas it is claimed for the palatinoids that, being hermetically sealed in jujube cachets, this decomposition cannot occur. The convenience of being able to prescribe Easton's syrup in a stable and constant dosage, as well as in so concentrated and elegant a form, will be appreciated by practitioners and patients alike.

### INVALIDS' BORDEAUX AND BURGUNDY

(Societé Viticola Francaise, Westminster Chambers, 5, Victoria Street, London, S.W.)

We have lately had samples of the above wines submitted to our notice. From trial that we have made of them we can safely recommend them to the notice of invalids who require picking up during the stages of convalescence. The wines are of the purest description, well matured, and exceedingly pleasant.

